# Genome-Wide Association Study of CSF Levels of 59 Alzheimer's Disease Candidate Proteins: Significant Associations with Proteins Involved in Amyloid Processing and Inflammation

**DOI:** 10.1371/journal.pgen.1004758

**Published:** 2014-10-23

**Authors:** John S. K. Kauwe, Matthew H. Bailey, Perry G. Ridge, Rachel Perry, Mark E. Wadsworth, Kaitlyn L. Hoyt, Lyndsay A. Staley, Celeste M. Karch, Oscar Harari, Carlos Cruchaga, Benjamin J. Ainscough, Kelly Bales, Eve H. Pickering, Sarah Bertelsen, the Alzheimer's Disease Neuroimaging Initiative, Anne M. Fagan, David M. Holtzman, John C. Morris, Alison M. Goate

**Affiliations:** 1 Department of Biology, Brigham Young University, Provo, Utah, United States of America; 2 Department of Psychiatry, Washington University School of Medicine, St Louis, Missouri, United States of America; 3 Hope Center for Neurological Disorders, Washington University School of Medicine, St Louis, Missouri, United States of America; 4 The Genome Institute, Washington University School of Medicine, St Louis, Missouri, United States of America; 5 Neuroscience Research Unit, Worldwide Research and Development, Pfizer Inc., Groton, Connecticut, United States of America; 6 Knight Alzheimer's Disease Research Center, Washington University School of Medicine, St Louis, Missouri, United States of America; 7 Department of Neurology, Washington University School of Medicine, St Louis, Missouri, United States of America; 8 Department of Developmental Biology, Washington University School of Medicine, St Louis, Missouri, United States of America; 9 Department of Pathology and Immunology, Washington University School of Medicine, St Louis, Missouri, United States of America; 10 Department of Genetics, Washington University School of Medicine, St Louis, Missouri, United States of America; University of California Los Angeles, United States of America

## Abstract

Cerebrospinal fluid (CSF) 42 amino acid species of amyloid beta (Aβ42) and tau levels are strongly correlated with the presence of Alzheimer's disease (AD) neuropathology including amyloid plaques and neurodegeneration and have been successfully used as endophenotypes for genetic studies of AD. Additional CSF analytes may also serve as useful endophenotypes that capture other aspects of AD pathophysiology. Here we have conducted a genome-wide association study of CSF levels of 59 AD-related analytes. All analytes were measured using the Rules Based Medicine Human DiscoveryMAP Panel, which includes analytes relevant to several disease-related processes. Data from two independently collected and measured datasets, the Knight Alzheimer's Disease Research Center (ADRC) and Alzheimer's Disease Neuroimaging Initiative (ADNI), were analyzed separately, and combined results were obtained using meta-analysis. We identified genetic associations with CSF levels of 5 proteins (Angiotensin-converting enzyme (ACE), Chemokine (C-C motif) ligand 2 (CCL2), Chemokine (C-C motif) ligand 4 (CCL4), Interleukin 6 receptor (IL6R) and Matrix metalloproteinase-3 (MMP3)) with study-wide significant p-values (p<1.46×10^−10^) and significant, consistent evidence for association in both the Knight ADRC and the ADNI samples. These proteins are involved in amyloid processing and pro-inflammatory signaling. SNPs associated with ACE, IL6R and MMP3 protein levels are located within the coding regions of the corresponding structural gene. The SNPs associated with CSF levels of CCL4 and CCL2 are located in known chemokine binding proteins. The genetic associations reported here are novel and suggest mechanisms for genetic control of CSF and plasma levels of these disease-related proteins. Significant SNPs in ACE and MMP3 also showed association with AD risk. Our findings suggest that these proteins/pathways may be valuable therapeutic targets for AD. Robust associations in cognitively normal individuals suggest that these SNPs also influence regulation of these proteins more generally and may therefore be relevant to other diseases.

## Author Summary

The use of quantitative endophenotypes from cerebrospinal fluid has led to the identification of several genetic variants that alter risk or rate of progression of Alzheimer's disease. Here we have analyzed the levels of 58 disease-related proteins in the cerebrospinal fluid for association with millions of variants across the human genome. We have identified significant, replicable associations with 5 analytes, Angiotensin-converting enzyme, Chemokine (C-C motif) ligand 2, Chemokine (C-C motif) ligand 4, Interleukin 6 receptor and Matrix metalloproteinase-3. Our results suggest that these variants play a regulatory role in the respective protein levels and are relevant to the inflammatory and amyloid processing pathways. Variants in associated with ACE and those associated with MMP3 levels also show association with risk for Alzheimer's disease in the expected directions. These associations are consistent in cerebrospinal fluid and plasma and in samples with only cognitively normal individuals suggesting that they are relevant in the regulation of these protein levels beyond the context of Alzheimer's disease.

## Introduction

Cerebrospinal fluid (CSF) contains promising biomarkers for neurological and psychiatric diseases such as Alzheimer's disease (AD), schizophrenia, and Parkinson's disease [1]–[4]. The brain directly and rapidly influences the composition of CSF, and as such, CSF analytes may provide insights into neurological and psychiatric disease pathways that may not be identifiable using blood or other biological fluids.

The use of endophenotypes in genome-wide association studies (GWAS) has provided novel insights into pathways and proteins that are associated with AD onset and progression [5], [6]. We have demonstrated the utility of CSF amyloid-beta (Aβ), apolipoprotein E (ApoE) and tau levels as endophenotypes for genetic studies of AD [7]–[16]. In our most recent work we used nearly 1,300 samples to conduct a GWAS with CSF tau levels [9]. In that study, Cruchaga et al. identified three genome-wide significant loci, including rs9877502, which also showed a consistent association with AD risk, tangle pathology, and global cognitive decline in independent datasets. The success of these and other similar efforts has led to broader efforts to develop and leverage datasets of this type [17]–[27].

While amyloid plaques and neurofibrillary tangles are the primary pathological features of AD, genetic, clinical, and animal studies demonstrate that endocytosis, cholesterol metabolism, and inflammatory and immune responses also play an important roles in AD pathogenesis [28]. To further leverage the advantages of the endophenotype based approach, we have sought to use analytes related to these other aspects of AD pathology. For this work we have obtained data from the Rules Based Medicine, Inc. (RBM) (Austin, TX) Human DiscoveryMAP Panel. This panel includes over 175 analytes selected from the constellation of known cytokines, chemokines, metabolic markers, hormones, growth factors, tissue remodeling proteins, angiogenesis markers, acute phase reactants, cancer markers, kidney damage markers, and central nervous system biomarkers. Analytes were quantitatively measured in CSF samples from 574 samples (including both cognitively normal and demented individuals) from two independent datasets to identify novel phenotypes that may contribute to the pathogenesis of AD. After careful quality control and evaluation of the literature we selected 59 AD-related analytes for analysis in genome-wide association studies. We identified genome-wide significant associations between putatively functional SNPs and five phenotypes (Angiotensin-converting enzyme (ACE), Chemokine (C-C motif) ligand 2 (CCL2), Chemokine (C-C motif) ligand 4 (CCL4), Interleukin 6 receptor (IL6R) and Matrix metalloproteinase-3 (MMP3)). The genetic basis of variance in these important disease-related analytes may provide insights into the mechanisms contributing to AD and other human diseases.

## Results

**Figure 1:**
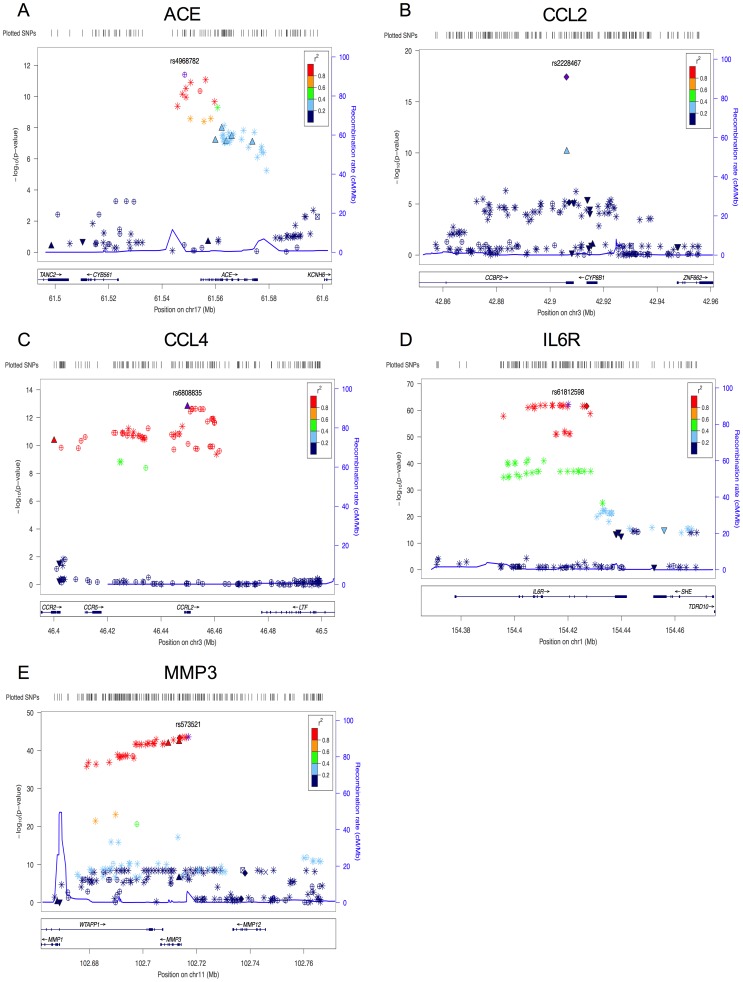
Plots for the region surrounding the genome-wide significant locus for each phenotype. A) region of rs4968782 (*ACE*), B) region of rs2228467 (*CCL2*), C) region of rs6808835 (*CCL4*), D) region of rs61812598 (*IL6R*), E) region of rs573521 (*MMP3*). The top SNP for each region is shown in purple. The correlations (r^2^) of each of the surrounding SNPs to the top SNP are shown in the indicated colors. Recombination rate is shown in blue. SNP annotation is as follows: circle = framestop, square-splice, diamond = nonsynonymous, triangle = coding, inverted triangle = UTR, X = conserved transcription factor binding, square with X = MCS44Placental, star = no annotation, circle with crosshairs = none.

From the combined GWAS on 59 phenotypes with connections to AD in our literature search (table 1) and with 5.8M SNPs (table 1), we identified 335 SNPs associated with five CSF phenotypes where p<1.47×10^−10^ (Bonferroni correction for 342 million tests) and other filtering criteria (see Methods: Statistical Analysis) were met (Table S1). At least one study-wide significant marker was directly genotyped (not imputed) for each locus that showed association. While there were other genome-wide significant associations with several of the other 54 phenotypes, these signals did not meet the additional filtering criteria. At least one SNP met the study-wide significance level (1.46×10^−10^) for each of the five phenotypes discussed in detail below (Table 2). All associations were robust to adjustment for APOE ε*4* genotype and CDR. In addition, all associations were qualitatively stable in clinical case/control strata as well as in strata approximating presence or absence of amyloid deposition based on CSF Aβ42 levels (Table 3). The most significant marker for each phenotype, all SNPs in the associated regions with putative function, and markers with previous records in the National Human Genome Research Institute (NHGRI) catalog of published genome-wide association studies (downloaded November 19^th^, 2013; http://www.genome.gov/gwastudies/) are listed in Table 2. P-values of all SNPs in the region surrounding each significant association are plotted in Figure 1. Manhattan plots for the five phenotypes can be found in supplementary figures S1-S5. Detailed descriptions of each gene can be found in Text S1.

**Table 1:** Analyte names.

Human Discovery MAP v1.0 Name	Official Protein Name	Official Gene Name
Adiponectin	Adiponectin (ADIPO)	Adiponectin (ADIPOQ)
Alpha-1 antitrypsin (AAT)	Alpha-1-antitrypsin (A1AT)	Serpin peptidase inhibitor, clade A (alpha-1 antiproteinase, antitrypsin), member 1 (SERPINA1)
Alpha-1-microglobulin (A1M)	Alpha-1-microglobulin (AMBP)	Alpha-1-microglobulin/bikunin precursor (AMBP)
Alpha-2-Macroglobulin (A2M)	Alpha-2-macroglobulin (A2M)	Alpha-2-macroglobulin (A2M)
Angiopoietin-2 (ANG-2)	Angiopoietin-2 (ANG-2)	Angiopoietin 2 (ANGPT2)
Angiotensin-converting enzyme (ACE)	Angiotensin I-converting enzyme (ACE)	Angiotensin I-converting enzyme (ACE)
Apolipoprotein A-I (Apo A-I)	Apolipoprotein A-I (Apo A-I)	Apolipoprotein A-I (APOA1)
Apolipoprotein D (Apo D)	Apolipoprotein D (Apo D)	Apolipoprotein D (APOD)
Apolipoprotein E (Apo E)	Apolipoprotein E (Apo E)	Apolipoprotein E (APOE)
Beta-2-microglobulin (B2M)	Beta-2-microglobulin (B2MG)	Beta-2-microglobulin (B2M)
C-reactive protein (CRP)	C-reactive protein (CRP)	C-reactive protein (CRP)
CD 40 antigen (CD40)	CD40 protein (CD40)	CD40 molecule (CD40)
Chemokine CC-4 (HCC-4)	C-C motif chemokine 16 (CCL16)	Chemokine (C-C motif) ligand 16 (CCL16)
Chromogranin-A (CgA)	Chromogranin-A (CgA)	Chromogranin A (CHGA)
Clusterin (CLU)	Clusterin (CLU)	Clusterin (CLU)
Complement C3 (C3)	Complement component 3 (C3)	Complement component 3 (C3)
Cortisol	Hydrocortisone	Brain-derived neurotrophic factor (BDNF)
Cystatin-C	Cystatin-C (CYTC)	Cystatin C (CST3)
Fas ligand (FasL)	Tumor necrosis factor ligand superfamily member 6 (TNFL6)	Fas ligand (TNF superfamily, member 6) (FASLG)
Fatty Acid-Binding Protein, heart (H-FABP)	Fatty acid-binding protein, heart (H-FABP)	Fatty acid binding protein 3, muscle and heart (FABP3)
Ferritin (FRTN)	Ferritin heavy chain (FRIH)/light chain (FRIL)	Ferritin, heavy polypeptide 1 (FTH1)/light polypeptide (FTL)
Fibrinogen	Fibrinogen alpha chain (FIBA)/beta chain (FIBB)/gamma chain (FIBG)	Fibrinogen alpha chain (FGA)/beta chain (FGB)/gamma chain (FGG)
Follicle Stimulating Hormone (FSH)	Glycoprotein hormones alpha chain (GLHA)/Follitropin subunit beta (FSHG)	Glycoprotein hormones, alpha polypeptide (CGA)/follicle stimulating hormone, beta polypeptide (FSHG)
Hepatocyte growth factor (HGF)	Hepatocyte growth factor (HGF)	Hepatocyte growth factor (HGF)
Insulin like growth factor binding protein 2 (IGFBP-2)	Insulin-like growth factor-binding protein 2 (IBP2)	Insulin-like growth factor binding protein 2 (IGFBP2)
Intercellular adhesion molecule 1 (ICAM-1)	Intercellular adhesion molecule 1 (ICAM1)	Intercellular adhesion molecule 1 (ICAM1)
Interferon gamma Induced Protein 10 (IP-10; CXCL10)	C-X-C motif chemokine 10 (CXL10)	Chemokine (C-X-C motif) ligand 10 (CXCL10)
Interleukin 3 (IL-3)	Interleukin 3 (IL3)	Interleukin 3 (IL3)
Interleukin-16 (IL-16)	Pro-interleukin-16 (IL16)	Interleukin 16 (IL16)
Interleukin-6 receptor (IL-6R)	Interleukin-6 receptor subunit alpha (IL6RA)	Interleukin 6 receptor (IL6R)
Interleukin-8 (IL-8)	Interleukin-8 (IL8)	Interleukin 8 (IL8)
Lectin-like oxidized low-density lipoprotein receptor 1 (LOX-1)	Oxidized low-density lipoprotein receptor 1 (OLR1)	Oxidized low density lipoprotein (lectin-like) receptor 1 (OLR1)
Leptin	Leptin (LEP)	Leptin (LEP)
Macrophage Inflammatory Protein-1 beta (MIP-1 beta)	Chemokine (C-C motif) ligand 4 (CCL4)	Chemokine (C-C motif) ligand 4 (CCL4)
Macrophage migration inhibitory factor (MIF)	Macrophage migration inhibitory factor (MIF)	Macrophage migration inhibitory factor (glycosylation-inhibiting factor) (MIF)
Matrix metalloproteinase-2 (MMP-2)	72 kDa type IV collagenase (MMP2)	Matrix metallopeptidase 2 (gelatinase A, 72 kDa gelatinase, 72 kDa type IV collagenase) (MMP2)
Matrix Metalloproteinase-3 (MMP-3)	Matrix metalloproteinase 3 (MMP3)	Matrix metallopeptidase 3 (MMP3)
Monocyte Chemotactic Protein 1 (MCP-1)	Chemokine (C-C motif) ligand 2 (CCL2)	Chemokine (C-C motif) ligand 2 (CCL2)
Myoglobin	Myoglobin (MYG)	Myoglobin (MG)
N-terminal prohormone of brain natriuretic peptide (NT proBNP)	Natriuretic peptides B (ANFB)	Natriuretic peptide B (NPPB)
Osteopontin (OPN)	Osteopontin (OPN)	Secreted phosphoprotein 1 (SPP1)
Pancreatic polypeptide (PPP)	Pancreatic prohormone (PAHO)	Pancreatic polypeptide (PPY)
Plasminogen Activator Inhibitor 1 (PAI-1)	Plasminogen activator inhibitor 1 (PAI1)	Serpin peptidase inhibitor, clade E (nexin, plasminogen activator inhibitor type 1), member 1 (SERPINE1)
Prolactin (PRL)	Prolactin (PRL)	Prolactin (PRL)
S100 calcium-binding protein B (S100-B)	Protein S100-B (S100B)	S100 calcium binding protein B (S100B)
Secretory Immunoglobulin A (IgA)	Immunoglobulin A (IgA)	CD79a molecule, immunoglobulin-associated alpha (CD79A)
Serum Amyloid P-Component (SAP)	Serum amyloid P-component (SAMP)	Amyloid P component, serum (APCS)
Serum glutamic oxaloacetic transaminase (SGOT)	Aspartate transaminase (AST)	glutamic-oxaloacetic transaminase 1, soluble (GOT1)
Sex hormone-binding globulin (SHBG)	Sex hormone-binding globulin (SHBG)	Sex hormone-binding globulin (SHBG)
Sortilin-1	Sortilin (SORT)	Sortilin 1 (SORT1)
Stem Cell Factor (SCF)	Kit ligand (SCF)	KIT ligand (KITLG)
T-Cell-Specific Protein RANTES (RANTES; CCL5)	C-C motif chemokine 5 (CCL5)	chemokine (C-C motif) ligand 5 (CCL5)
Tissue inhibitor of metalloproteinases 1 (TIMP-1)	Metalloproteinase inhibitor 1 (TIMP1)	TIMP metallopeptidase inhibitor 1 (TIMP1)
Transforming growth factor alpha (TGF-alpha)	Transforming growth factor alpha (TGFA)	Transforming growth factor, alpha (TGFA)
Tumor necrosis factor receptor 2 (TNFR2)	Tumor necrosis factor receptor superfamily member 1B (TNR1B)	Tumor necrosis factor receptor superfamily, member 1B (TNFRSF1B)
Tumor necrosis factor-related apoptosis-inducing ligand receptor 3 (TRAIL-R3)	Tumor necrosis factor receptor superfamily member 10C (TR10C)	Tumor necrosis factor receptor superfamily, member 10c, decoy without an intracellular domain (TNFRSF10C)
Vascular cell adhesion molecule-1 (VCAM-1)	Vascular cell adhesion protein 1 (VCAM1)	Vascular cell adhesion molecule 1 (VCAM1)
Vascular endothelial growth factor (VEGF)	Vascular endothelial growth factor A (VEGFA)	Vascular endothelial growth factor A (VEGFA)
von Willebrand factor (vWF)	von Willebrand factor (vWF)	von Willebrand factor (vWF)

The name of each “AD-related” analyte in the Human Discovery MAP panel, official protein name and official structural gene name are presented.

**Table 2:** Selected study-wide significant results.

Phenotype	SNP	CHR	BP	Non-effect allele	Effect allele	ADNI	Knight ADRC	Combined	Effect Direction	ADNI r^2^	MAF	Nearest Gene	Function	Regulome Score
ACE	rs4968782	17	61548476	a	g	4.60E-08	1.59E-05	3.94E-12	+	0.11	0.3802	ACE	intergenic	7
ACE	rs4459609	17	61548948	a	c	1.62E-07	1.15E-04	1.09E-10	+	0.09	0.3748	ACE	intergenic	7
ACE	rs4316	17	61562309	t	c	8.67E-09	2.47E-02	1.10E-08	+	0.14	0.4634	ACE	Synonymous C81C	2a
ACE	rs4343	17	61566031	a	g	6.26E-08	2.16E-02	3.71E-08	+	0.11	0.4903	ACE	Synonymous G606G	2b
CCL2	rs2228467	3	42906116	a	g	6.30E-12	7.42E-08	3.71E-18	+	0.13	0.061	CCBP2	Nonsynonymous T122G	5
CCL4	rs6808835	3	46449864	t	g	5.35E-10	2.97E-05	1.59E-13	−	0.12	0.1513	CCRL2	Synonymous G330G	7
CCL4	rs6762266	3	46452863	t	c	0.00004074	5.349E-10	2.27E-13	+	0.12	0.1505	CCRL2	intergenic	1f
CCL4	rs11575821	3	46422355	a	g	6.42E-04	1.129E-09	1.22E-11	−	0.10	0.1468	CCR5	intergenic	1f
CCL4	rs113263161	3	46425718	a	g	0.00007856	2.696E-08	1.41E-11	−	0.10	0.1383	CCR5	intergenic	2b
CCL4	rs11574428	3	46446721	a	t	0.0001202	1.649E-08	1.48E-11	−	0.10	0.1384	CCRL2	intergenic	1f
CCL4	rs3092960	3	46400062	a	g	0.00005041	1.661E-07	4.43E-11	−	0.10	0.1331	CCR2	synonymous T348T	2b
CCL4	rs6441977	3	46450072	a	g	1.13E-07	1.13E-04	7.66E-11	−	0.10	0.1387	CCRL2	Nonsynonymous G538A	5
IL6R	rs61812598	1	154420087	a	g	2.61E-37	1.45E-27	5.91E-63	−	0.40	0.4047	IL6R	intronic	7
IL6R	rs4845622	1	154411419	a	c	2.28E-36	4.44E-28	1.36E-62	+	0.39	0.4086	IL6R	intronic	7
IL6R	rs2228145	1	154426970	a	c	1.30E-37	1.11E-26	2.70E-62	+	0.40	0.4043	IL6R	Nonsynonymous A1073C	7
IL6R	rs4129267	1	154426264	a	g	1.30E-37	1.11E-26	2.70E-62	−	0.40	0.4043	IL6R	intronic	2b
IL6R	rs3811448	1	154516578	a	g	4.90E-07	8.24E-10	3.36E-15	+	0.09	0.1947	TDRD10	Nonsynonymous G643A	5
IL6R	rs2229238	1	154437896	a	g	2.07E-08	2.20E-07	2.29E-14	+	0.11	0.1924	IL6R	UTR3	4
MMP3	rs573521	11	102716980	g	a	3.00E-26	7.12E-20	2.39E-44	+	0.31	0.4832	MMP3	intergenic	5
MMP3	rs645419	11	102716321	g	a	4.15E-26	7.12E-20	3.26E-44	+	0.30	0.4836	MMP3	intergenic	7
MMP3	rs679620	11	102713620	g	a	6.36E-26	7.12E-20	4.93E-44	+	0.30	0.4843	MMP3	Nonsynonymous A133C	7
MMP3	rs7926920	11	102698724	g	a	1.32E-25	1.57E-18	2.56E-42	+	0.30	0.4854	WTAPP1	ncRNA_intronic	6
MMP3	rs11225434	11	102691482	t	c	4.94E-24	7.38E-17	4.50E-39	−	0.29	0.4947	WTAPP1	ncRNA_intronic	5
MMP3	rs948399	11	102771140	c	t	9.612E-07	8.93E-06	4.29E-11	−	0.07	0.286	MMP12	intergenic	2b
MMP3	rs495366	11	102695108	g	a	1.84E-06	7.73E-06	6.19E-11	−	0.07	0.2784	WTAPP1	ncRNA_intronic	7
MMP3	rs650108	11	102708787	g	a	8.29E-07	2.68E-05	1.01E-10	−	0.07	0.2729	MMP3	intronic	7
MMP3	rs603050	11	102680229	c	t	4.39E-06	9.86E-05	1.84E-09	−	0.06	0.2897	WTAPP1	ncRNA_intronic	NA

This table includes the following SNPs for each phenotype with significantly associated variants: the most significant marker, all significant SNPs with putative function, Regulome scores of 1 or 2, and all SNPs that have previous records in the NHGRI catalog of published genome-wide association studies (downloaded November 19th, 2013). For each marker the chromosome (CHR), base pair position (BP), Alleles (Non-effect allele, effect allele), p-value from the ADNI dataset (ADNI), p-value from the Knight ADRC dataset (Knight ADRC), combined sample p-value (Combined), direction of association with respect to the effect allele (Effect Direction), variance explained by the marker in the discovery series (ADNI r^2^), minor allele frequency (MAF), Nearest Gene, Functional annotation (Function), and Regulome Score is provided.

**Table 3:** Association of top hits with additional phenotypes of interest.

Phenotype	SNP	CHR	BP	Plasma Analyte P-alue	Amyloid deposition strata	No amyloid deposition strata	CDR = 0	CDR>0	AD association P-alue (BETA; SE)	AB42	Tau	pTau	Other associated phenotypes
IL6R	rs4845622	1	154411419	5.55E-02	6.43E-05	3.29E-09	2.93E-03	5.97E-01	0.93 (−.0014, 0.016)	0.88	0.89	0.82	None
IL6R	rs61812598	1	154420087	2.67E-09	5.28E-04	5.38E-01	6.01E-02	1.57E-02	0.76 (−.0048, 0.016)	0.70	0.65	0.72	None
IL6R	rs4129267	1	154426264	4.64E-07	1.73E-02	2.83E-01	9.59E-01	1.20E-02	0.097 (0.0006, 0.016)	0.63	0.54	0.64	Fibrinogen, Asthma, C-eactive protein, IL6R (blood), Pulmonary function
IL6R	rs2228145	1	154426970	4.64E-07	1.73E-02	2.83E-01	9.59E-01	1.20E-02	0.85 (0.0032, 0.017)	0.62	0.60	0.69	None
IL6R	rs2229238	1	154437896	1.46E-11	6.57E-07	2.04E-08	1.51E-09	2.38E-07	0.55 (0.012, 0.020)	0.17	0.67	0.83	Coronary heart disease
IL6R	rs3811448	1	154516578	7.91E-12	9.52E-07	2.36E-09	2.13E-10	1.09E-07	0.11 (0.031, 0.019)	0.04	0.29	0.86	None
CCL2	rs2228467	3	42906116	4.82E-02	1.98E-08	2.72E-10	5.36E-16	2.25E-03	0.52 (−.021, 0.033)	0.14	0.51	0.80	Monocyte count
CCL4	rs3092960	3	46400062	2.36E-05	4.04E-06	2.00E-06	7.60E-07	2.65E-05	0.6789 (0.0094, 0.023)	0.60	0.89	0.90	None
CCL4	rs11575821	3	46422355	2.42E-04	2.99E-06	1.05E-04	2.99E-07	1.14E-05	0.79 (0.0063, 0.024)	0.46	0.98	0.96	None
CCL4	rs113263161	3	46425718	1.20E-04	6.40E-06	6.74E-07	7.85E-07	5.09E-06	0.83 (0.0052, 0.024)	0.53	0.85	0.74	None
CCL4	rs11574428	3	46446721	2.82E-04	4.51E-06	4.80E-05	3.67E-07	1.54E-05	0.88 (0.0034, 0.023)	0.55	0.93	0.68	None
CCL4	rs6808835	3	46449864	1.96E-04	2.86E-07	3.07E-07	8.13E-09	8.88E-06	0.81 (0.0050, 0.025)	0.64	0.53	0.47	None
CCL4	rs6441977	3	46450072	6.68E-04	7.26E-06	2.01E-04	7.63E-07	3.63E-05	0.81 (0.0056, 0.023)	0.80	0.59	0.41	None
CCL4	rs6762266	3	46452863	1.59E-04	1.84E-07	6.84E-07	1.25E-08	8.88E-06	0.70 (0.0093, 0.025)	0.59	0.64	0.58	None
MMP3	rs603050	11	102680229	2.68E-01	3.69E-03	1.18E-04	1.78E-07	3.95E-03	0.40 (0.015, 0.018)	0.90	0.67	0.75	None
MMP3	rs11225434	11	102691482	2.92E-02	1.69E-12	3.20E-05	6.12E-05	5.67E-14	0.091 (−.027, 0.016)	0.86	0.91	0.94	Matrix metalloproteinase 1 levels (blood)
MMP3	rs495366	11	102695108	4.66E-01	6.07E-03	2.25E-09	2.32E-07	2.50E-04	0.29 (0.018, 0.017)	0.92	0.78	0.64	Matrix metalloproteinase 1 levels (blood)
MMP3	rs7926920	11	102698724	4.89E-02	1.90E-13	1.22E-07	1.52E-07	1.95E-14	0.053 (−.029, 0.015)	0.99	0.76	0.74	None
MMP3	rs650108	11	102708787	5.62E-01	3.46E-03	1.33E-08	1.77E-07	2.66E-04	0.31 (0.018, 0.017)	0.95	0.59	0.63	None
MMP3	rs679620	11	102713620	4.44E-02	6.05E-15	9.95E-08	1.54E-09	3.07E-14	0.046 (−.032, 0.016)	0.96	0.68	0.51	None
MMP3	rs645419	11	102716321	4.44E-02	4.10E-15	9.95E-08	1.02E-09	3.07E-14	0.039 (−.032, 0.015)	0.96	0.68	0.50	None
MMP3	rs573521	11	102716980	4.44E-02	3.40E-15	9.95E-08	8.12E-01	3.07E-14	0.038 (−.032, 0.016)	0.93	0.67	0.50	None
MMP3	rs948399	11	102771140	1.37E-02	1.78E-07	2.28E-05	5.42E-07	6.35E-05	0.74 (0.0057, 0.017)	0.76	0.18	0.23	None
ACE	rs4968782	17	61548476	7.93E-06	4.06E-04	8.39E-09	1.61E-08	1.54E-04	0.0073 (−.044,0.016)	0.57	0.72	0.83	None
ACE	rs4459609	17	61548948	9.70E-06	1.17E-03	5.83E-08	2.25E-07	2.05E-04	0.0066 (−.044, 0.016)	0.55	0.37	0.79	None
ACE	rs4316	17	61562309	2.04E-02	3.97E-04	9.60E-06	1.98E-05	2.22E-04	0.0038 (−.045, 0.016)	0.82	0.13	0.65	None
ACE	rs4343	17	61566031	3.77E-05	5.62E-04	5.10E-05	2.16E-05	1.26E-03	0.0048 (−.044, 0.015)	0.71	0.02	0.85	Angiotensin-converting enzyme activity

Results of association with respective plasma analytes, CSF analytes levels in samples with low AB42 (evidence of amyloid deposition), those with high AB42 (no evidence of deposition) is shown for each SNP from table 2. In each case the direction of the association is consistent with the original association reported in Table 2. Also shown is results of association with AD status (including BETA and Standard Error, from IGAP Stage 1 analysis [30]; the sign of Beta has been adjusted to reflect the Effect Allele in Table 3 for each marker as appropriate), CSF AB42 levels, CSF Tau levels and CSF pTau levels (p-values from Cruchaga et al 2013 [9]) are shown. Finally, other phenotypes for which the SNP has shown genome-wide significance in the NHGRI GWAS catalog are presented.

### Effects of age and gender

We observed a significant association between CSF MMP3 and CCL2 and gender, where both analytes were lower in females relative to males in cognitively normal samples from both ADNI and the Knight ADRC. We also observed significantly increased MMP3 and CCL2 levels with increasing age in cognitively normal samples from both ADNI and the Knight ADRC. We failed to detect consistent association in both the ADNI and Knight ADRC samples with plasma levels of these analytes and age or gender. Full results including slopes of the regression models can be found in table S2 (CSF results) and table S3 (plasma results).

### Association results

#### Angiotensin-converting enzyme (ACE)

We identified seven SNPs significantly associated with ACE levels in CSF (Figure 1A). The minor allele of rs4968782 was associated with higher ACE CSF protein levels (p = 3.94×10^−12^) and explains 11% of the variance in CSF ACE levels. The signal was consistent in cases/controls and in CSF Aβ42 strata defining presence/absence of Aß pathology and was also observed between this SNP and plasma ACE levels (p = 7.93×10^−16^). A synonymous substitution in the *ACE* gene, rs4343 (rs4968782/rs4343: r^2^ = 0.93; D′ = 1.0), was also associated with ACE levels in CSF and plasma (p = 3.71×10^−8^; p = 1.10×10^−8^, respectively). CSF and plasma levels of ACE showed a significant and moderate correlation (Pearson's correlation coefficient = 0.28, p = 4.86×10^−6^). Another synonymous substitution, rs4316 is also in high LD with these markers and shows significant association with CSF and plasma levels of ACE (see table 3). Both rs4343 and rs4316 are predicted as “likely to affect binding transcription factors” by RegulomeDB. None of these SNPs remained significant after conditioning upon the others. Rs4343 also appears in the NHGRI GWAS catalog associated with increased ACE activity levels in serum [29]. Association with AD status was observed (all four SNPs p<0.0075) in 17,008 AD cases and 37,154 controls from the International Genomics of Alzheimer's Project (IGAP) as described by Lambert et al and accessible at http://www.pasteur-lille.fr/en/recherche/u744/igap/igap_download.php [30]. For each of the four ACE SNPs, rs4968782, rs4459609, rs4316 and rs4343, the same allele was associated with increased ACE levels and decreased risk for AD (table 3).

#### Chemokine (C-C motif) ligand 2 (CCL2) also known as monocyte chemotactic protein 1

We identified one SNP, rs2228467, which results in a non-synonymous change (V41A) in the chemokine binding protein-2 (*CCBP2*) that is associated with increased CCL2 protein levels (CSF p = 3.71×10^−18^). This marker accounts for 13% of the variance in CSF CCL2 levels. Other SNPs in this region with moderate linkage disequilibrium (LD) also showed strong, but not study-wide significant, associations (Figure 1B). The association of rs2228467 with CSF levels of CCL2 remained significant in cases/controls and in CSF Aβ42 strata defining presence/absence of Aβ pathology and was nominally associated with CCL2 levels in plasma (table 3). CSF and plasma CCL2 levels were significantly correlated (Pearson's correlation coefficient = 0.23, p = 1.93×10^−4^). Both SIFT and PolyPhen2 predicted the rs2228467 V41A change to be damaging. The IGAP analysis failed to detect association between rs2228467 and risk for AD (table 3) [30]. We failed to detect association between CSF CCL2 levels and AD status (p = 0.90). This SNP was not associated with other phenotypes in the NHGRI GWAS catalog.

#### Chemokine (C-C motif) ligand 4 (CCL4) also known as macrophage inflammatory protein 1 beta

We identified 66 polymorphisms associated with CCL4 levels in CSF. This is a trans effect, all associated SNPs are located within a 187 kb region of chromosome 3 surrounding the Chemokine (C-C Motif) Receptor-Like 2 *(CCRL2)* gene (figure 1C). The most significant association was observed with rs6808835 (p = 1.59×10^−13^). A non-synonymous SNP in the *CCRL2* gene, rs6441977 (rs6808835/rs6441977: r^2^ = 0.93; D′ = 1.0), results in a V180M substitution and shows significant association with CSF CCL4 levels in the combined sample (p = 7.66×10^−11^) with CCL4 levels decreasing with each copy of the minor allele. This SNP explains 10% of the variance in CSF CCL4 levels. Several significant, intergenic markers in this same region are predicted as “likely to affect transcription factor binding and gene expression” (rs6762266, rs11575821, rs11574428) and “likely to affect transcription factor binding” (rs113263161, rs3092960) using RegulomeDB. None of these SNPs remained significant after conditioning upon the others. In addition to the association in the total dataset, this SNP shows consistent association in cases/controls, in CSF Aβ42 strata defining presence/absence of Aβ pathology and with CCL4 levels in plasma (table 3). CSF and plasma levels of CCL4 were significantly correlated (Pearson's correlation coefficient = 0.37, p = 6.59×10^−10^). SIFT and PolyPhen 2 predicted the amino acid change to be benign. No SNPs associated with CCL4 showed significant association with AD in the IGAP analysis (table 3) and no significant SNPs were associated with other phenotypes in the NHGRI GWAS catalog.

#### Interleukin 6 receptor (IL6R)

We identified 176 SNPs significantly associated with soluble IL6R (sIL6R) levels in CSF. These significant SNPs were located in the region of chromosome 1 surrounding the *TDRD12*, *SHE*, and *IL6R* genes (figure 1D). The most significant association was with rs61812598 (p = 5.9×10^−62^). Two non-synonymous SNPs were also associated with increased sIL6R protein levels in both CSF and plasma: rs2228145 (gene = *IL6R*, D358A, CSF p = 2.70×10^−62^, plasma p = 4.64×10^−67^) and rs3811448 (gene = *TDRD10*, V215I, CSF p = 3.36×10^−15^, plasma p = 7.91×10^−12^). CSF and plasma levels of sIL6R showed a significant correlation (Pearson's correlation coefficient = 0.49, p = 2.20×10^−16^). In addition, rs2228145 exhibits nearly perfect LD with rs61812598 (r^2^ = 0.99; D′ = 1.0). Rs3811448 has a high D′ with both rs2228145 (D′ = 0.96) and rs61812598 (D′ = 0.96) but its r^2^ with these SNPs is low (r^2^ = 0.15 with both SNPs). Another significant SNP in this region, rs4129267, has nearly complete LD with both rs61812598 and rs2228145 (D′>0.99, r^2^>0.99 in both cases), and is predicted by RegulomeDB as “likely to affect transcription factor binding.” Tests conditioning on any of these SNPs results in no significant associations in this region. Associations with both rs2228145 and rs61812598 (essentially the same test as they exhibit nearly perfect LD) remained genome-wide significant when conditioning upon rs3811448. Rs2228145 accounts for about 40% of variance in CSF sIL6R levels. Neither amino acid change is predicted to affect its respective protein's function by SIFT or Polyphen 2. The IGAP analysis failed to detect association between the markers associated with sIL6R and AD risk (table 3) [30]. We found significant SNPs in our analyses that have been previously reported to be associated with asthma [31], C-reactive protein levels (rs4129267) [32], and coronary heart disease (rs2229238) [33] in the NHGRI GWAS catalog.

#### Matrix metalloproteinase-3 (MMP3)

Eighty-five SNPs were significantly associated with MMP3 levels (figure 1E). The most significant association was observed with rs573521 (p = 2.39×10^−44^). A non-synonymous mutation in *MMP3*, rs679620 (K45E), shows high LD with rs573521 (r^2^ = 0.99; D′ = 1.0). Neither of these two SNPs remained significant after conditioning upon the other. Rs679620 was also significantly associated with MMP3 levels in CSF (p = 4.93×10^−44^) with MMP3 levels increasing with each copy of the minor allele. This SNP accounts for about 30% of the variance in CSF MMP3 levels. Association of this marker is consistent in cases/controls and in CSF Aβ42 strata defining presence/absence of Aβ pathology and was also observed nominally with plasma MMP3 levels (table 3). CSF and plasma MMP3 levels were moderately and significantly correlated (Pearson's correlation coefficient = 0.33, p = 2.06×10^−5^). SIFT and PolyPhen 2 predicted the rs679620 K45E amino acid change to be benign. Rs948399 also showed significant association and was predicted by RegulomeDB as “likely to affect transcription factor binding.” Three SNPs, rs573521, rs645419 and rs679620 showed nominal significance in the IGAP AD association analysis (table 3). From the NHGRI GWAS catalog, five SNPs previously reported to be associated with MMP1 levels in plasma in a recent genome-wide association study (rs7926920, rs11225434, rs495366, rs603050, and rs650108) [34] are also significantly associated with MMP3 levels in CSF in our data. MMP3 and MMP1 are located in the same region on chromosome 11 and show strong correlation in their pattern of expression [35], [36] suggesting that there may be coordinated regulation of these two genes. These SNPs are located in the 37 kb region between MMP3 and MMP1 and show high LD (D′∼1) with rs573521 and rs679620.

#### Multivariate analysis of top SNPs

We used Multiphen, to perform a multivariate test of the linear combination of phenotypes most associated with the genotypes at each of our top SNPs [37]. At least one marker from each of the loci in our top hits showed genome-wide significance in the joint models (Table 4). Several SNPs showed strong association with sIL6R and prolactin (PRL). Rs2228467, which showed primary association with CCL2, also showed association with glutamic-oxaloacetic transaminase 1 (GOT1). Full results including p-values and metaanalysis of each of the top SNPs with each phenotype are provided in Table S4.

**Table 4:** Multiphen analysis.

SNP	CHR	BP	Primary	Knight ADRC	ADNI	Analytes
rs4845622	chr1	154411419	IL6R	3.35E-54	8.56E-71	PRL
rs61812598	chr1	154420087	IL6R	4.12E-56	2.38E-81	PRL
rs4129267	chr1	154426264	IL6R	9.56E-55	7.41E-82	PRL
rs2228145	chr1	154426970	IL6R	9.56E-55	7.41E-82	PRL
rs2229238	chr1	154437896	IL6R	1.34E-10	1.86E-10	N/A
rs3811448	chr1	154516578	IL6R	3.17E-12	5.37E-11	N/A
rs2228467	chr3	42906116	CCL2	4.72E-09	4.19E-16	GOT1
rs3092960	chr3	46400062	CCL4	2.91E-07	8.33E-08	N/A
rs11575821	chr3	46422355	CCL4	3.47E-07	1.02E-09	N/A
rs113263161	chr3	46425718	CCL4	2.24E-07	7.70E-08	N/A
rs11574428	chr3	46446721	CCL4	9.59E-06	2.27E-09	N/A
rs6808835	chr3	46449864	CCL4	1.65E-05	2.67E-11	N/A
rs6441977	chr3	46450072	CCL4	1.04E-05	3.10E-08	N/A
rs6762266	chr3	46452863	CCL4	2.15E-05	2.67E-11	N/A
rs603050	chr11	102680229	MMP3	5.36E-07	2.36E-10	N/A
rs11225434	chr11	102691482	MMP3	3.64E-27	1.82E-42	N/A
rs495366	chr11	102695108	MMP3	8.37E-10	9.67E-13	N/A
rs7926920	chr11	102698724	MMP3	6.92E-29	1.67E-46	N/A
rs650108	chr11	102708787	MMP3	5.06E-09	3.40E-12	N/A
rs679620	chr11	102713620	MMP3	8.58E-31	1.14E-48	N/A
rs645419	chr11	102716321	MMP3	8.58E-31	8.54E-49	N/A
rs573521	chr11	102716980	MMP3	8.58E-31	3.46E-48	N/A
rs948399	chr11	102771140	MMP3	4.28E-11	9.87E-10	N/A
rs4968782	chr17	61548476	ACE	3.93E-10	7.09E-12	N/A
rs4459609	chr17	61548948	ACE	3.19E-10	3.31E-12	N/A
rs4316	chr17	61562309	ACE	5.04E-06	3.91E-17	N/A
rs4343	chr17	61566031	ACE	3.98E-06	4.46E-19	N/A

For each SNP the rs number (SNP, the chromosome (CHR), base pair position (BP) primary associated analyte (Primary), p-value from joint phenotype testing in the Knight ADRC and ADNI samples, and the additional associated phenotypes (Secondary) after Bonferroni correction for 28 SNPs (alpha = 0.0018) is shown.

## Discussion

We have identified loci significantly associated with levels of five AD-related CSF analytes. Our findings include cis effects (defined as SNPs within 5 kb on either side of the transcribed gene) for ACE, sIL6R and MMP3 levels and trans effects for CCL2 and CCL4 levels. For each of the loci except ACE, a non-synonymous SNP is among the most strongly associated variants. SNPs in all five loci were associated with each of the analytes even after a highly conservative multiple test correction (alpha = 1.46×10^−10^). All results are consistent when analyses are stratified by clinical status and when stratified by CSF Aβ42 levels that are indicative of AD pathology, suggesting that these results are relevant in normal conditions, not just in the context of AD. In addition, all of the associated SNPs showed association with their respective plasma analytes, further supporting the robustness of the genetic associations. The ACE, MMP3 and CCL2 proteins have been previously described to have an impact on amyloid beta processing. The remaining proteins are involved in the pro-inflammatory response. The results of our Multiphen analysis did not provide strong evidence that these SNPs regulate expression of multiple traits in similar pathways.

### Angiotensin-converting enzyme (ACE)

Angiotensin converting enzyme (ACE) is encoded by the *ACE* gene (17q23.3) and has been previously implicated in AD pathogenesis. *In vitro*, ACE inhibits Aβ aggregation in an activity-dependent manner by slowing the rate of fibril formation [38]–[40]. ACE may inhibit Aβ aggregation by converting the highly amyloidogenic Aβ42 peptide into the more stable Aβ40 peptide [41]. *In vivo*, inhibition of ACE activity in an AD mouse model promotes Aβ42 deposition in the hippocampus [41]. Studies in mouse and human brain homogenate demonstrate that ACE causes Aβ degradation in a two-step process. First, ACE cleaves Aβ42 into Aβ40 and then Aβ40 undergoes degradation [41].

ACE activity is elevated in the CSF of AD patients [42]. Neuroblastoma cells exposed to synthetic Aβ42 oligomers, but not monomeric Aβ42, produce elevated ACE protein levels and ACE activity, suggesting that Aβ aggregates may stimulate the up-regulation of ACE in AD brains as a mechanism of combatting the accumulation of these protein aggregates.

Our findings suggest that SNPs within the *ACE* locus alter CSF and plasma ACE levels. Several SNPs in the ACE gene region have significant associations with increased levels of ACE. Conditional analyses suggest a single signal in this region is responsible for the association. While we are unable to determine the specific functional allele in this region, rs4343 and rs4316 are in nearly perfect LD with each other (D′ = 0.99, r^2^ = 0.91) and both are inferred to have regulatory function (table 3). Rs4316 has not been studied in AD or reported to be associated with other phenotypes to date. Rs4343 is a widely studied marker in ACE, and we found a significant association of this SNP with increased CSF ACE levels. The minor allele “G” of rs4343 has been previously reported to be significantly associated with increased plasma ACE activity levels and plasma ACE protein levels [21], [29]. Our observation of increased CSF ACE levels with the G allele of rs4343 is consistent with these reports.

In relation to AD, the findings with rs4343 have been varied with some studies suggesting association with CSF Aβ42 levels and AD risk while others do not [14], [43]–[49]. Recent data from 1269 samples with CSF and genetic data failed to detect association between CSF Aβ42, Tau or pTau_181_ levels and these SNPs in ACE (table 3) [9]. Results from the recent International Genomics of Alzheimer's Project provide evidence of association between the SNPs in *ACE* we report to be associated with higher CSF ACE levels and reduced risk for AD [30]. In addition, recent work using 600 samples with CSF ACE measurements found a significant increase in ACE levels with increasing ptau/Aβ42 ratio (which was used as a predictor of AD status) [50], further reinforcing the relationship between ACE levels and AD status.

Our results demonstrate in human subjects that SNPs in the *ACE* gene are associated with elevated CSF and plasma ACE protein levels and that these SNPs are also associated with reduced AD risk. Taken with *in vitro* and *in vivo* studies that demonstrate that ACE cleaves and clears Aβ in an activity-dependent manner, these findings suggest that individuals carrying polymorphisms that increase ACE protein, and possibly ACE activity, may be better able to clear accumulating Aβ aggregates and are thus at reduced risk for developing AD.

### Matrix metalloproteinase-3 (MMP3)

Matrix metalloproteinase 3 (encoded by *MMP3*; located on 11q22.3) is hypothesized to contribute to endogenous, physiologic clearance of amyloid plaques. MMP3 is expressed in neurons, astrocytes, microglia and vascular cells [51]. MMP3 is preferentially localized in senile plaques in the parietal cortex of AD brains, while hippocampal plaques are relatively spared of MMP3 [52]. Aβ treatment causes upregulation of MMP3 expression in primary astrocyte cultures and in mixed hippocampal cultures [53]. MMP3 also degrades extracellular Aβ [54]. The closely related MMP2 and MMP9 proteins have been well studied in the context of AD pathogenesis. Astrocytes that surround plaques in AD mouse brains show enhanced MMP2 and MMP9 expression [55]. Conditioned astrocyte media is sufficient to reduce synthetic Aβ levels, which is abolished with treatment of inhibitors specific to MMP2 and MMP9 [55]. MMP9 can degrade fibrillar Aβ in vitro and plaques in hippocampal slice cultures [52], [56], [57]. In an AD mouse model, knocking out MMP2 and MMP9 results in increased steady-state Aβ [55]. Thus, MMP proteins may contribute to Aβ clearance by promoting Aβ catabolism.

Supporting the relationship of MMP3 with Alzheimer's disease, CSF MMP3 levels are increased in individuals with a ptau_181_/Aβ42 ratio indicative of AD [50]. In a previous study, we failed to detect an association between significant SNPs in this study and CSF Aβ42, Tau or pTau_181_ levels (table 3) [9]. Studies testing the association of *MMP3* SNPs and haplotypes and risk for AD have produced mixed results [58]–[60]. There appears to be two tiers of association in this locus, one group of SNPs with p-values less than 1E-38, which includes the non-synonymous SNP rs679620, and another which p-values between 1E-08 and 1E-12, including several variants with inferred regulatory function. Conditional analyses of our data in this region indicate that the more strongly associated group of variants tags a single association signal and that no independent associations are detected in this region. While we cannot definitively identify the causal variant, rs679620, a non-synonymous SNP in the *MMP3* region, was significantly associated with increased CSF MMP3 levels. This suggests a possible protective effect of this variant with respect to AD. We found that rs573521, rs645419 and rs679620 are associated with increased CSF MMP3 levels in this study and with reduced risk of AD in the IGAP study. These associations provide additional support for the role of MMP3 in AD pathology. In addition, the identification of SNPs in the intergenic region between *MMP3* and *MMP1* that are associated with both MMP3 and MMP1 levels suggest a common regulatory locus or close functional relationship between these members of the *MMP* gene family. The identification of SNPs near *MMP3* that are associated with increased CSF MMP3 protein levels and reduced AD risk supports the protective role of MMP3 in clearing Aβ from human brains.

### Chemokine (C-C motif) ligand 2 (CCL2)

CCL2, also called monocyte chemotactic protein-1 or MCP-1, is encoded by the *CCL2* gene, located on chromosome 17q11.2-q12. It is a chemokine that is involved in immunoregulatory and pro-inflammatory processes. Amyloid plaques in AD brains are surrounded by activated immune cells that produce CCL2 among other chemokines [61]. In the absence of CCL2, amyloid pathology is accelerated in an AD mouse model, illustrating its important role in amyloid plaque clearance and pointing to a potentially reparative role in AD [62]. However, overexpression of CCL2 in an AD mouse model resulted in marked accumulation of reactive microglia and enhanced diffuse plaque accumulation, suggesting a role in Aβ aggregation [63]. *In vitro* work suggests that inhibition of CCL2 synthesis, reduces Aβ25–35- and Aβ1–42-induced toxicity in primary neuronal cultures [64]. Interestingly, treatment of primary astrocyte cultures with synthetic Aβ42 causes astrocytes to increase CCL2 synthesis and release [64] and astrocyte migration in response to CCL2 is reduced in the presence of Aβ42 [65]. Thus, Aβ42 in AD brains may stimulate astrocyte-mediated CCL2 release and result in increased neuronal susceptibility to Aβ42 toxicity. The immune system involves a delicate and perfectly coordinated balance to function well; so, it is conceivable that CCL2 could play reparative and deleterious roles in AD pathogenesis.

A 2006 study evaluated CCL2 levels in serum samples from 48 individuals with Mild Cognitive Impairment (MCI), 94 AD patients and 24 age-matched controls [66]. Significantly increased plasma CCL2 levels were found in MCI and mild AD, but not in severe AD patients, as compared with controls. It has also been reported that increased CSF CCL2 levels at baseline in patients with prodromal AD correlated with a faster cognitive decline during the study's follow-up period [67]. The largest study to date examined 600 samples with CSF CCL2 measurements and observed a significant increase in CCL2 protein levels with ptau/Aβ42 ratio indicative of AD [50]. As is the case with most pro-inflammatory cytokines and cytokine receptors, the levels increase in AD cases.

Rs2228467, located within the *CCBP2* gene, is significantly associated with increased CSF CCL2 protein levels. The *CCBP2* gene encodes the chemokine-binding protein 2 and demonstrates a high affinity for binding to CCL2 [68]. Conditional analyses suggest that this marker accounts for the entirety of the association signal in this region. Previous studies suggest that chemokine receptors can demonstrate high affinity binding to chemotactic proteins [68]; however, how polymorphisms in one chemokine affect expression and function of associated chemokines is poorly understood. PolyPhen2 and SIFT both predicted this amino acid change to be damaging. A recent study found that rs2228467 is significantly associated with lower circulating monocyte counts in the blood (p = 1.57×10^−7^) [69]. If the rs2228467 polymorphism affects the chemokine function of CCBP2 or CCL2, then this could have downstream effects on the recruitment of macrophages and dendritic cells and, in turn, monocyte development.

CCL2 is known to be a necessary component in monocytes crossing the blood brain barrier [61], [70]. Our findings suggest that increased CCL2 associated with variation at rs2228467 may cause a chemotactic response that results in lower levels of circulating monocytes in the blood. While rs2228467 has strong effects on CCL2 levels and CSF CCL2 levels change in Alzheimer's disease, this SNP does not appear to impact risk for AD or CSF Aβ42 levels (p = 0.45) [9]. However, as CCL2 has been implicated in the pathogenesis of diseases characterized by monocytic infiltrates, like psoriasis [71], rheumatoid arthritis [72] and atherosclerosis [73] further investigation of rs2228467 with regard to these and related diseases is clearly warranted.

Here we have demonstrated in human subjects that SNPs in the *CCBP2* gene are significantly associated with elevated CSF CCL2 protein levels. While, CSF CCL2 protein levels are not significantly associated with AD risk, evidence in mouse and cell models of AD suggest that increasing CCL2 levels increases microgliosis, amyloid plaque accumulation, and neuronal toxicity associated with Aβ. Taken together, these findings implicate CCBP2 and CCL2 as risk factors for AD pathogenesis.

### Chemokine (C-C motif) ligand 4 (CCL4)

The CCL4 protein is encoded by the *CCL4* gene (17q12). Studies evaluating the relationship between AD and inflammation have shown that CCL4 is expressed in subpopulations of reactive astrocytes and in microglia [74]. Neuritic plaques in AD are surrounded by activated microglia and astrocytes, which may produce inflammatory products when stimulated with Aβ [75]. Macrophages showed an increased secretion of CCL4 when treated with Aβ. Current information concerning CCL4 and other plaque-associated chemokines suggests that their production plays a role in the recruitment and accumulation of astrocytes and microglia in senile plaques [76]. These data suggest a possible relationship between CCL4 and AD pathogenesis.

We identified association between several SNPs, including one non-synonymous SNP, one synonymous SNP and five markers with predicted regulatory effects, and CSF CCL4 levels. Conditional analyses suggest that these SNPs tag a single association signal in this region. These SNPs do not show association with AD in the IGAP dataset or with CSF Aβ42, Tau or pTau_181_ levels (table 3). In addition, CSF CCL4 levels are not significantly associated with ptau_181_/Aβ42 ratio, a predictor of AD status [50]. These results do not indicate a role for CCL4 levels in risk for AD. However, CCL4 may play a role in HIV Type 1 transmission, AIDS disease progression, and acute kidney injury [77], [78]. Thus it will be important to evaluate the impact of rs6441977 (V168M polymorphism in *CCRL2*; associated with decreased CCL4 levels) and other markers with regulatory effects, on these and related diseases.

### Interleukin 6 receptor (IL6R)

The interleukin 6 receptor (IL6R) is a protein encoded by the *IL6R* gene (1q21). Interleukin 6 is a potent pleiotropic pro-inflammatory cytokine that regulates cell growth and differentiation and plays an important role in the immune response and may also play a role in hippocampal neurogenesis [79].

We identified association with sIL6R levels for several SNPs in the *IL6R* region. Conditional analyses suggest that this is a single signal is driven by rs61812598 and other SNPs in high LD with these markers. Among these, rs2228145, rs3811448 and rs4129267 have predicted functional effects (see table 3). Rs3811448, a non-synonymous marker in the associated region is not significant when conditioning upon rs61812598, rs4129267 or rs2228145. Conversely, both rs61812598 rs4129267 and rs2228145 remain highly significant upon conditioning for rs3811448. This makes it clear that there is a single signal in this region, tagged by rs61812598, rs4129267 and rs2228145, which are in complete LD with each other.

Rs2228145 is a non-synonymous polymorphism in the *IL6R gene*, (D358A). While both SIFT and Polyphen 2 predict this change to be benign, rs2228145 has recently been shown to significantly increase plasma concentrations of sIL-6R, and reduce concentrations of membrane-bound IL-6R, resulting in impaired IL-6 responsiveness [80]. These results demonstrate that consequential changes in protein levels, likely resulting from the rs2228145 polymorphism, may translate into a functional impairment in IL-6R signaling. The rs2228145 polymorphism and other SNPs in this region have previously been shown to be significantly associated with plasma sIL-6 levels [21], [81] Trans-signaling is important for IL6-mediated cellular communication with molecular targets and that blocking trans-signaling lessen the deleterious effects of IL6 signaling [79]. Trans-signaling occurs via sIL6R, which is the proteolyzed product of IL6R. IL6R is proteolyzed by γ-secretase, which also cleaves APP [82]. Thus, polymorphisms in IL6R that modify IL6 signaling may result in modulation of signaling to many downstream targets that directly or indirectly influence AD pathogenesis.

Additionally, rs2228145 has been implicated previously as significantly increasing the risk of sporadic AD in a Chinese Han population in subjects without the *APOE ε4* allele [83]. While sIL6R levels have been previously reported to decrease in AD cases [84], a recent study using a much larger sample found a significant increase in CSF sIL6R levels and increasing ptau_181_/Aβ42 ratio [50], suggesting a possible relationship between sIL6R levels and AD pathogenesis.

It has been proposed that there is a reciprocal relationship between IL-6 and Aβ. The IL-6/sIL-6R complex is reported to enhance *APP* transcription and expression [85]–[87]. Based on these data, rs2228145, which is associated with increased sIL6R levels, would be predicted to alter CSF Aβ42 levels and risk for AD. Unfortunately, we did not detect association of markers in *IL6R* with AD risk in the IGAP analysis and association with AD biomarkers was weak and inconsistent (table 3).

Rs4129267 is located within the intronic region of *IL6R*. This SNP is inferred to be “likely to affect binding” of the Olf-1 transcription factor using RegulomeDB. Like rs2228145, this marker is associated with sIL6R levels [21], [81]. In addition, this marker has been reported to be associated with levels of fibrinogen and C-reactive protein in blood as well as asthma and pulmonary function [31], [32], [88]–[90]. While it remains unclear what the causal marker is for this association signal due to the high levels of LD, the putative functional effects of both rs2228145 and rs4129267 make them top candidates for future investigation.

Dysregulated production of IL6 and its receptor are implicated in the pathogenesis of many diseases, including multiple myeloma [91], autoimmune diseases [92], and prostate cancer [93]. In addition, the association of several significant SNPs in our study with asthma, C-reactive protein levels and coronary heart disease highlights the relationship between the inflammatory response and these disorders. This information suggests a central role for the IL6/sIL6R complex in these and possibly other diseases and suggests that further characterization of the effects of rs2228145 and rs4129267 on human disease phenotypes is warranted.

In conclusion, we have identified significantly associated SNPs for five different AD-related analytes. These associations are robust across different biological fluids, dementia status and inferred presence of AD pathology both within and between independent sample sets. The SNPs observed to be associated with CSF ACE and MMP3 levels also appear to show association with AD in the predicted direction, providing support for previous hypotheses of involvement of these genes and their function in amyloid clearance for risk for AD. While inflammation is known to play an important role in AD, the pro-inflammatory markers investigated here, and their associated SNPs, do not appear to alter AD risk or disease progression. However, because the immune system is an exceedingly complex set of signaling cascades that must be perfectly regulated in order to function properly and because this regulation involves constant flux, we may not be able to fully capture the subtle effects in the function of these proteins that have a cumulative effect on AD pathogenesis over the course of a lifetime. The reproducibility of our findings in cognitively normal individuals and in plasma levels of the respective proteins as well as putative functional effects of these variants suggest that these SNPs may directly affect their respective proteins. Thus, insights into the genetic basis of variance in important pro-inflammatory protein levels are relevant to other diseases that are modulated by those processes. Finally, our findings demonstrate the continued utility of an endophenotype-based approach to finding functional alleles and disease-associated loci.

## Methods

### Subjects

#### ADNI

Data used in the preparation of this article were obtained from the ADNI database (www.loni.ucla.edu\ADNI). CSF was collected as described previously [94]. Genetic and phenotypic data for 308 samples was available for this study. Demographics of the samples included in this manuscript are reported in Table 5.

**Table 5:** Sample characteristics.

	Knight ADRC	ADNI
N	266	308
Percent Female	60%	40%
Percent APOE e4 positive	33%	48%
Percent CDR = 0	35%	21%
Percent Amyloid positive*	45%	52%
Percent CDR = 0 and amyloid positive	36%	37%
Percent CDR> = 0.5 and amyloid positive	72%	84%
Mean CSF ACE Levels ng/ml (SD)	3.26 (1.23)	0.306 (0.16)**
Mean CSF CCL2 Levels pg/ml (SD)	682 (183)	2.72 (0.13)**
Mean CCL4 Levels pg/ml (SD)	17.7 (7.14)	1.22 (0.20)**
Mean CSF IL6R Levels ng/ml (SD)	1.13 (0.34)	0.001 (0.15)**
Mean CSF MMP3 Levels ng/ml (SD)	0.10 (0.065)	−0.47 (0.20)**

For samples from the Knight Alzheimer's Disease Research Center and Alzheimer's Disease Neuroimaging Initiative the number of samples, percent female, percent of APOE e4 carriers, percent of non-demented (CDR = 0) samples, percent amyloid positive, percent of CDR = 0 samples that are amyloid positive, percent of CDR> = 0.5 samples that were amyloid positive) and mean and standard deviation of the key analytes from this study are shown.

*Amyloid positivity is inferred from CSF AB42 levels (KADRC AB42<500 pg/ml; ADNI AB42<192 pg/ml).

**ADNI phenotypes were obtained from ADNI after transformation to approximate a normal distribution.

#### Knight ADRC

CSF samples from 266 individuals from the Knight-Alzheimer's disease Research Center at Washington University School of Medicine (Knight ADRC) were used in this study. A detailed description of these samples and CSF collection methods has been published previously [9], [95]. Demographics of these samples are described in Table 5.

### Phenotypes

CSF and plasma samples from the Knight ADRC were evaluated for levels of 190 analytes using the Human DiscoveryMAP Panel and a Luminex 100 platform. CSF and plasma samples from the ADNI sample were assessed using the same Human DiscoveryMAP Panel and measurement platform [21], [94]. After filtering each set independently for phenotypes that had valid measurements in at least 90% of the samples, the intersection resulted in 76 analytes. For each of the 76 analytes that passed quality assurance in both datasets we performed a PubMed search on August 11, 2013. The purpose of this literature search was to reduce the phenotype list to those that are relevant to our AD centered samples from the Knight ADRC and ADNI, thus reducing the dimensionality of the data and concentrating statistical power on the most relevant phenotypes. Search terms included any of the following three terms, the name of the analyte on the chip, the official gene name and the official protein name *and* the term “Alzheimer's disease”. Analytes with more than 50 search results are considered to be “AD-related”. For analytes with fewer than 50 search results we inspected the manuscripts manually to determine whether there was evidence for a relationship with AD. A list of all analyte names on the chip along with official gene and protein names and results of the literature search is provided in supplementary Table S5.

### Genotyping

All samples were genotyped using the Illumina 610 or the Omniexpress chip. Prior to statistical analysis, sample data were filtered using rigid quality control (QC) criteria by array: minimum call rate (98%), minimum minor allele frequency (2%), and exclusion of SNPs out of Hardy-Weinberg equilibrium (p<1×10^−6^). Unanticipated duplicates and related individuals were prioritized after calculating pairwise genome-wide estimates of identity-by-descent. Eigensoft was used to calculate principal component factors for each sample and confirm ethnicity [96]. These calculations were included as covariates in our analysis to adjust for possible confounding effects of population stratification.

### Imputation

The 1000 genome data (June 2012 release) and the Beagle software were used to impute genotypes in the combined ADNI and Knight ADRC samples [97]. SNPs with a Beagle r^2^ of 0.3 or lower, a minor allele frequency (MAF) lower than 0.05, out of Hardy-Weinberg equilibrium (p<1×10^−6^), a call rate lower than 95% or a Gprobs score lower than 0.90 were removed. A total of 5,815,690 SNPs passed the QC process.

### Statistical analysis

The Kolmogorov-Smirnov goodness-of-fit test was performed to evaluate normality of the 59 phenotypes of interest in the Knight ADRC samples. When deviations were observed, phenotypes were log transformed to approximate a normal distribution. The ADNI data for these samples and phenotypes had already been adjusted to fit normal distribution patterns by the ADNI biomarker core. Associations reported for age and gender were performed in cognitively normal samples only to reduce potential confounds of dementia.

We performed a genome-wide association for each of the 59 phenotypes to identify genetic loci associated with protein levels in CSF. For the initial association analysis in each series we used PLINK to perform linear regression and evaluated the association between the additive model for 5.8M SNPs and each phenotype [98]. Age, gender, and the principal components from Eigensoft analysis were included as covariates. Variance explained by each marker is reported as the difference in the model r^2^ between full models with and without the SNP included as a variable. Association of SNPs of interest with plasma analyte levels in the ADNI and Knight ADRC samples was calculated using the same approach. Analysis of each sample separately reduces the possible confound of demographic or ascertainment differences between the ADNI and Knight ADRC samples.

Genome-wide association results from the two datasets were meta-analyzed using the default settings in METAL [99]. Genomic inflation factor scores (GIF) were estimated using the R package GenABEL [100]. We set a strict and extremely conservative study-wide alpha level of 1.46×10^−10^ for the combined analysis. This was calculated by applying a Bonferroni correction for 5.8 million SNPs and 59 analytes, or 342.2 million tests. SNPs that met the initial significance criteria were further filtered using the following criteria. First, we rejected SNPs where the direction of the effect was different in the Knight ADRC and ADNI datasets. Second, we removed all SNPs where the minor allele frequency was less than 5% (unless they were directly genotyped). Finally, we rejected all associations with phenotypes where the genomic inflation factor was greater than 1.03 (GIF was calculated without SNPs where MAF is <0.05). Conditional analyses on each of the genome-wide significant SNPs were conducted using the –condition function in PLINK. We also performed additional analyses in the genome-wide significant loci to determine the stability of the results when stratified by clinical AD status and by CSF AB42 levels. CSF AB42 strata were based on levels that approximate amyloid deposition detected in PET scans using Pittsburgh Compound B (PIB). For the KADRC samples values less than 500 pg/ml indicate PIB retention/Aβ deposition, while values greater than 500 pg/ml indicate PIB negativity and the absence of Aβ deposition [95]. For the ADNI samples values less than 192 pg/ml indicate retention/Aβ deposition, while values greater than 192 pg/ml indicate PIB negativity and the absence of Aβ deposition [101].

We used the R package Multiphen, which performs a multivariate test of the linear combination of phenotypes most associated with the genotypes at each SNP, to evaluate each of the top hits for joint effects on multiple phenotypes in the study [37]. Analysis was carried out in the KADRC and ADNI samples separately using default settings as described here (http://cran.at.r-project.org/web/packages/MultiPhen/vignettes/MultiPhen.pdf).

### GIF statistic for IL6R

Initial results of IL6R indicated a GIF statistic greater than 1.03 suggesting the p-values were inflated by confounding variables. By removing a 500 kb window on either side of the strongest signal we identified that the inflation was due to the large number of highly significant p-values surrounding the IL6R gene (adjusted GIF = 1.017). We did not remove this phenotype as it appears the inflation is due to a strong and replicable association signal in this single region. We analyzed other phenotypes that failed GIF quality control using the same strategy and did not observe similar phenomena.

### Association with risk for Alzheimer's disease

For each locus where association was detected with the CSF endophenotypes we obtained data from the International Genomics of Alzheimer's Project (IGAP) association study of AD Stage 1 results [30]. IGAP is a large two-stage study based upon genome-wide association studies (GWAS) in individuals of European ancestry. In stage 1, IGAP used genotyped and imputed data for 7,055,881 single nucleotide polymorphisms (SNPs) to meta-analyse four previously-published GWAS datasets consisting of 17,008 Alzheimer's disease cases and 37,154 controls (The European Alzheimer's disease Initiative – EADI the Alzheimer Disease Genetics Consortium – ADGC The Cohorts for Heart and Aging Research in Genomic Epidemiology consortium – CHARGE The Genetic and Environmental Risk in AD consortium – GERAD). In stage 2, 11,632 SNPs were genotyped and tested for association in an independent set of 8,572 Alzheimer's disease cases and 11,312 controls. Finally, a meta-analysis was performed combining results from stages 1 & 2.

### Bioinformatics analyses

We used ANNOVAR to annotate SNPs of interest with location and functional information [102]. RegulomeDB was used to annotate SNPs within known and predicted regulatory elements [103].

We used SIFT and POLYPHEN2 for preliminary assessments of the functional consequences of amino acid changes [104], [105].

All data collection was conducted under approval by the appropriate Institutional Review Boards. Analyses presented here were approved by the Institutional Review Board at Brigham Young University (E110252).

## Supporting Information

Figure S1 Manhattan plots for ACE. The x-axis shows each marker that was analyzed, sorted by chromosome and position. The y-axis shows the −log_10_ of the p-value for association with the respective phenotype.
(PDF)

Figure S2 Manhattan plots for CCL2. The x-axis shows each marker that was analyzed, sorted by chromosome and position. The y-axis shows the −log_10_ of the p-value for association with the respective phenotype.
(PDF)

Figure S3 Manhattan plots for CCL4. The x-axis shows each marker that was analyzed, sorted by chromosome and position. The y-axis shows the −log_10_ of the p-value for association with the respective phenotype.
(PDF)

Figure S4 Manhattan plots for IL6R. The x-axis shows each marker that was analyzed, sorted by chromosome and position. The y-axis shows the −log_10_ of the p-value for association with the respective phenotype.
(PDF)

Figure S5 Manhattan plots for MMP3. The x-axis shows each marker that was analyzed, sorted by chromosome and position. The y-axis shows the −log_10_ of the p-value for association with the respective phenotype.
(PDF)

Table S1 All study-wide significant variants. For each marker the chromosome (CHR), base pair position (BP), Alleles (Allele1, Allele2), p-value from the ADNI dataset (ADNI), p-value from the Knight ADRC dataset (Knight ADRC), combined sample p-value (Combined), direction of association (Direction), minor allele frequency (MAF), Genotyping status, function, Nearest Genes, exonic function, amino acid change and regulome score is provided.
(XLSX)

Table S2 Association of CSF ACE, IL6R, CCL4, MMP3 and CCL2 levels with age and gender. The slope and p-value for linear regression with age and gender is shown for the ADNI and KADRC samples. P-values of less than 0.05 are shown in bold.
(XLSX)

Table S3 Association of plasma ACE, IL6R, CCL4, MMP3 and CCL2 levels with age and gender. The slope and p-value for linear regression with age and gender is shown for the ADNI and KADRC samples. P-values of less than 0.05 are shown in bold.
(XLSX)

Table S4 Multiphen results. For each SNP and phenotype the p-value from ADNI, Knight ADRC, metaanalysis results are shown.
(XLSX)

Table S5 Analyte names. The name of each analyte in the Human Discovery MAP documentation, official protein name and official structural gene name and resuls of the PubMed search for previous links to Alzheimer's disease are presented.
(XLSX)

Text S1 Descriptions of each gene.
(DOCX)
