# Supplementary figures and images for: Genome-Wide Association Study of CSF Levels of 59 Alzheimer's Disease Candidate Proteins: Significant Associations with Proteins Involved in Amyloid Processing and Inflammation
Source: PLoS Genet. 2014 Oct 23;10(10):e1004758. doi: 10.1371/journal.pgen.1004758 (PMC4207667; doi:10.1371/journal.pgen.1004758)

ACE\_ManhattanPlot

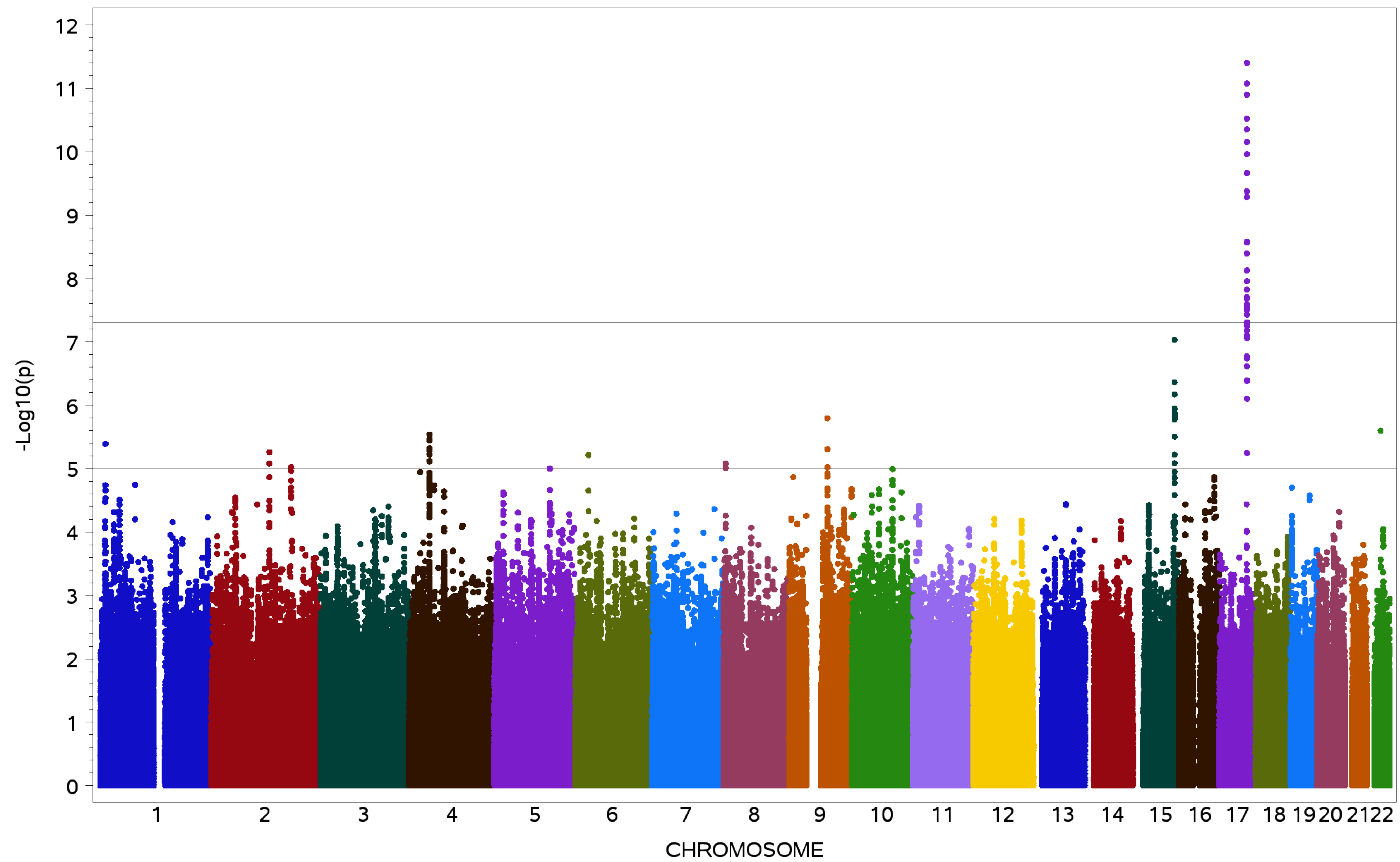

Supplement: Figure S1 — Manhattan plots for ACE. The x-axis shows each marker that was analyzed, sorted by chromosome and position. The y-axis shows the −log10 of the p-value for association with the respective phenotype. (PDF) [file pgen.1004758.s001.pdf]

MonocyteCP1\_ManhattanPlot

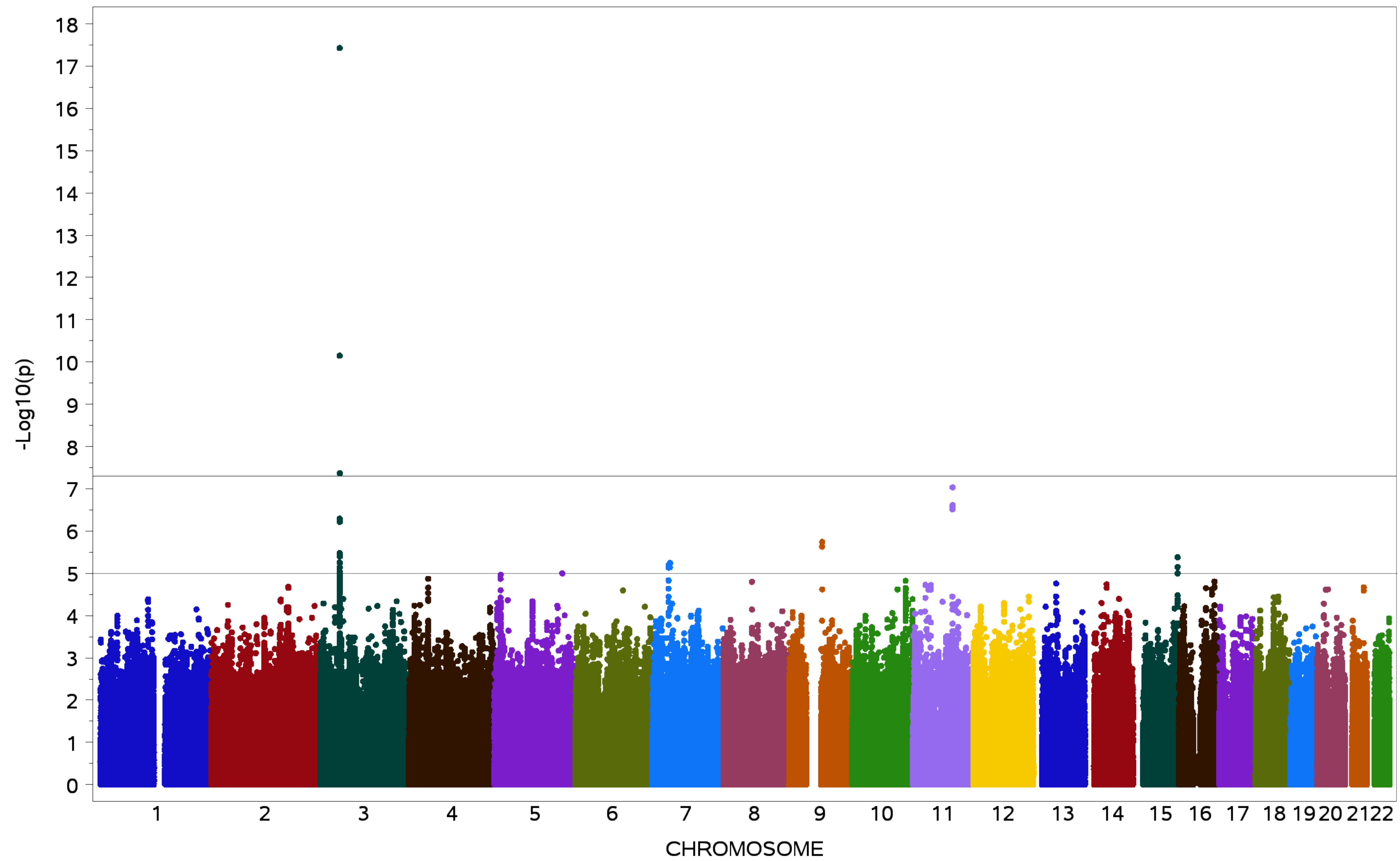

Supplement: Figure S2 — Manhattan plots for CCL2. The x-axis shows each marker that was analyzed, sorted by chromosome and position. The y-axis shows the −log10 of the p-value for association with the respective phenotype. (PDF) [file pgen.1004758.s002.pdf]

MIP1beta\_ManhattanPlot

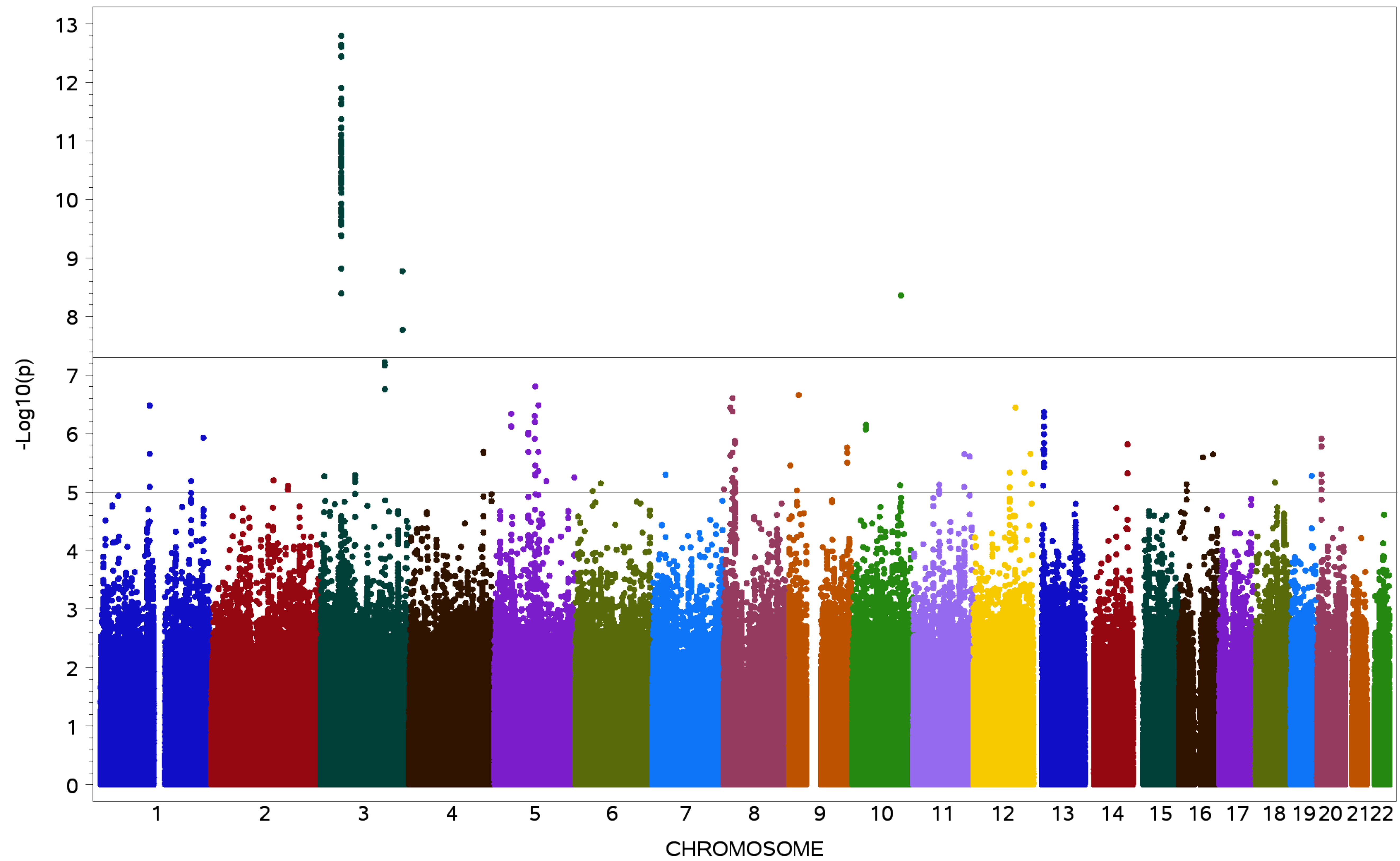

Supplement: Figure S3 — Manhattan plots for CCL4. The x-axis shows each marker that was analyzed, sorted by chromosome and position. The y-axis shows the −log10 of the p-value for association with the respective phenotype. (PDF) [file pgen.1004758.s003.pdf]

IL6R\_ManhattanPlot

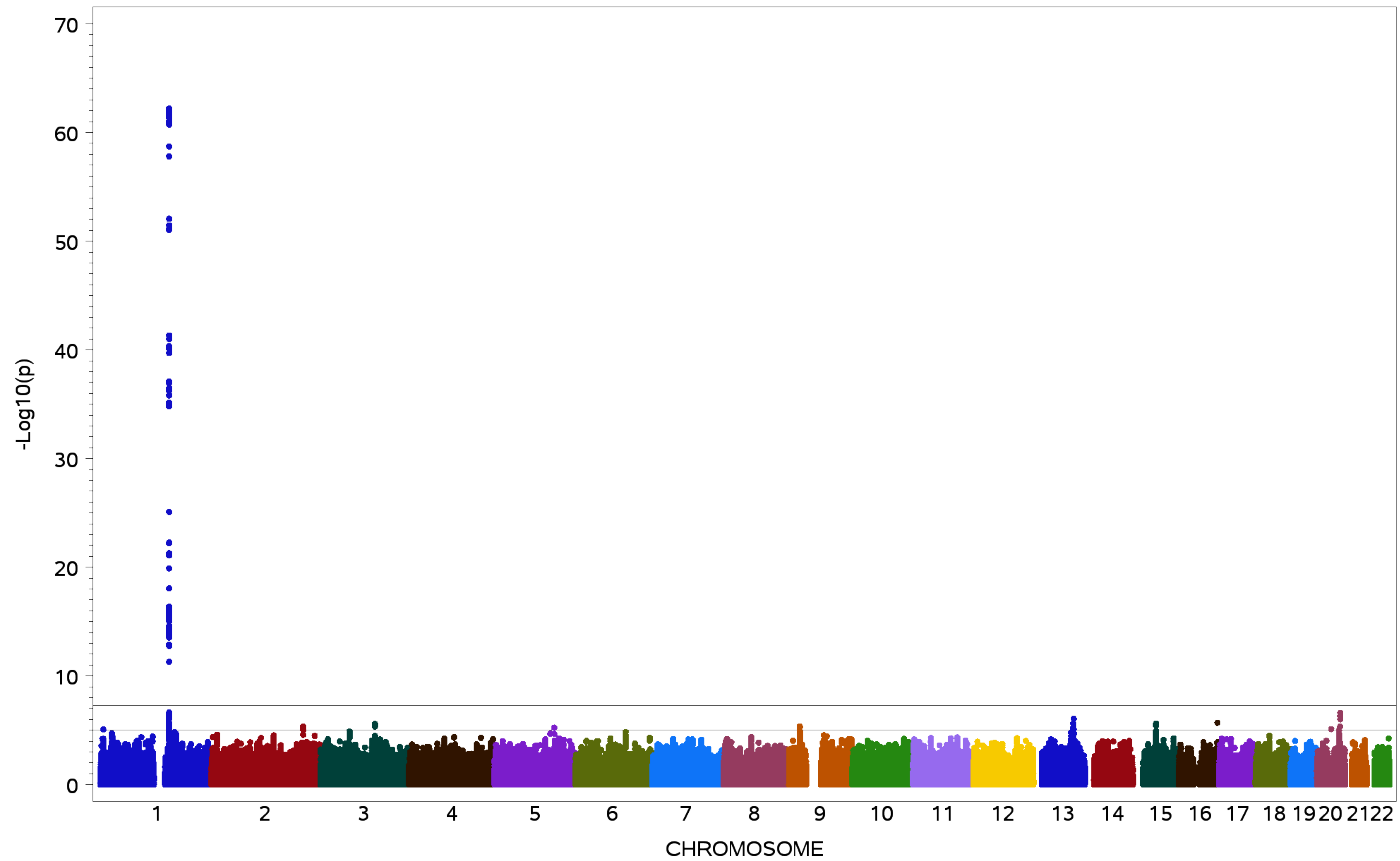

Supplement: Figure S4 — Manhattan plots for IL6R. The x-axis shows each marker that was analyzed, sorted by chromosome and position. The y-axis shows the −log10 of the p-value for association with the respective phenotype. (PDF) [file pgen.1004758.s004.pdf]

MMP3\_ManhattanPlot

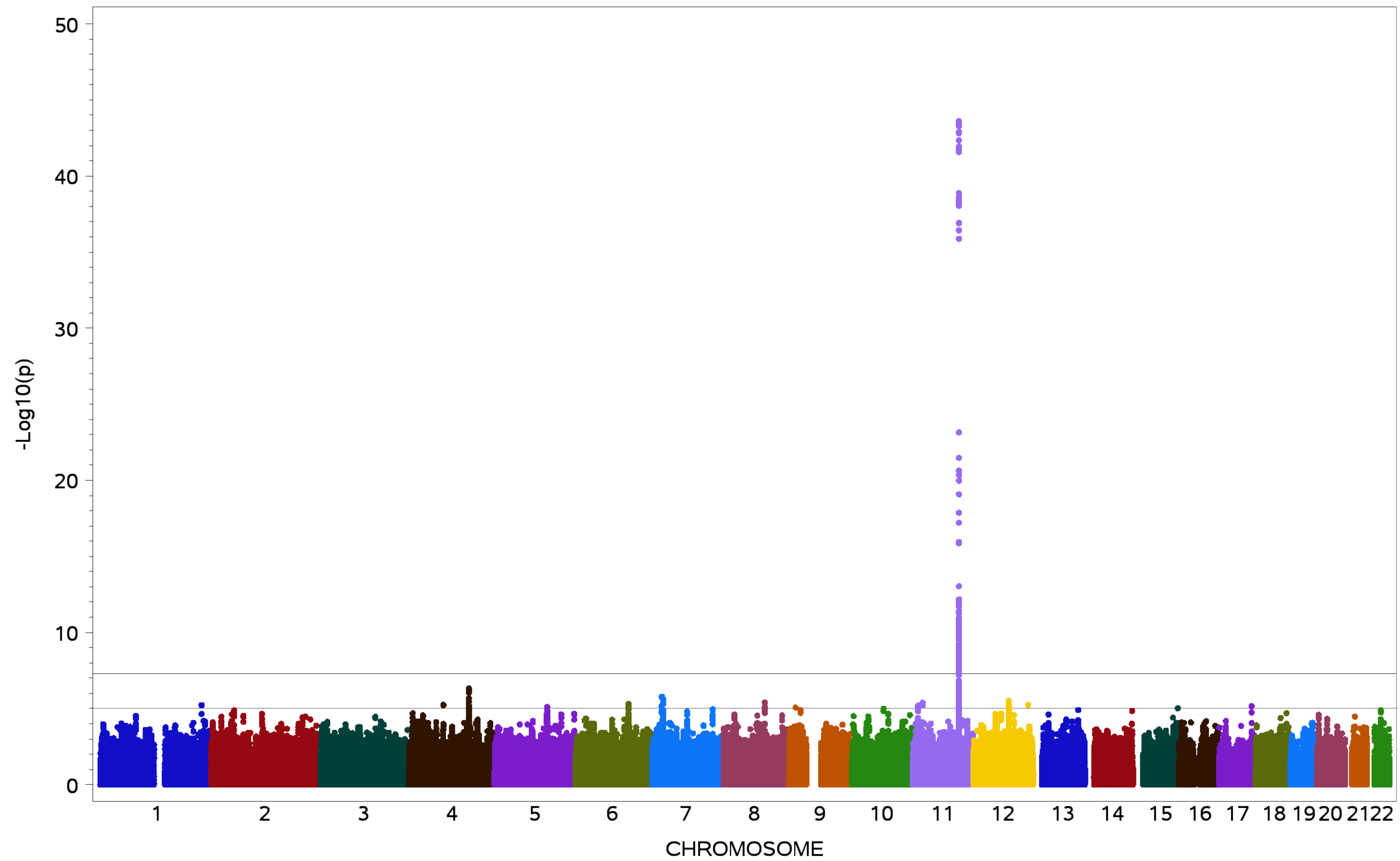

Supplement: Figure S5 — Manhattan plots for MMP3. The x-axis shows each marker that was analyzed, sorted by chromosome and position. The y-axis shows the −log10 of the p-value for association with the respective phenotype. (PDF) [file pgen.1004758.s005.pdf]
