# Supplementary material for: Genome-Wide Association Study of CSF Levels of 59 Alzheimer's Disease Candidate Proteins: Significant Associations with Proteins Involved in Amyloid Processing and Inflammation
Source: PLoS Genet. 2014 Oct 23;10(10):e1004758. doi: 10.1371/journal.pgen.1004758 (PMC4207667; doi:10.1371/journal.pgen.1004758)
Supplement: Text S1 — Descriptions of each gene. (DOCX) [file pgen.1004758.s011.docx]

Angiotensin converting enzyme (ACE) is encoded by the *ACE* gene (17q23.3) and is involved in catalyzing the conversion of angiotensin I into a physiologically active peptide angiotensin II. Angiotensin II is a potent vasopressor and aldosterone-stimulating peptide that controls blood pressure and fluid-electrolyte balance. ACE also plays a key role in the renin-angiotensin system. In addition to its role in blood pressure, ACE also plays a role in neuropeptide metabolism and immune response.

MMP3 is involved in the breakdown of extracellular matrix in normal physiological processes, such as embryonic development, reproduction, and tissue remodeling, as well as in disease processes, such as arthritis [[1](#_ENREF_1)] and metastasis [[2](#_ENREF_2)]. MMP3 degrades fibronectin, laminin, collagens III, IV, IX, and X, cartilage proteoglycans, and is involved in wound repair, progression of atherosclerosis, and tumor initiation. A common functional 5A/6A polymorphism in the MMP-3 gene promoter has been linked to ovarian cancer [[3](#_ENREF_3)], myocardial infarction [[4](#_ENREF_4)], carotid artery stenosis [[5](#_ENREF_5)], colorectal cancer [[6](#_ENREF_6)], and migraines [[7](#_ENREF_7)]. Human, mouse, and cell studies point to a role for matrix metalloproteinases in AD pathogethesis.

CCL2, also called monocyte chemotactic protein-1 or MCP-1, is encoded by the *CCL2* gene, located on chromosome 17q11.2-q12. Studies in mouse models demonstrate that it displays chemotactic activity for monocytes, basophils, and microglia, but not for neutrophils or eosinophils [[8](#_ENREF_8)]. In the brain, CCL2 is highly expressed in astrocytes and microglia [[9](#_ENREF_9)]. Neuronal expression of CCL2 occurs particularly in the hippocampus [[9](#_ENREF_9)]. CCL2 and its receptor are implicated in AD, multiple sclerosis and ischemic brain injury (reviewed in [[10](#_ENREF_10)]), where CCL2 expression is altered in human and animal models of these degenerative injuries [[11-16](#_ENREF_11)].

The CCL4 protein is encoded by the *CCL4* gene (17q12), and is a mitogen-inducible pro-inflammatory monokine that is one of the major HIV-suppressive factors produced by CD8+ T-cells. CCL4 is a small polypeptide that regulates cell trafficking of various types of leukocytes, and plays fundamental roles in the development, homeostasis, and function of the immune system. CCL4 is a ligand for CCR4. This chemokine is associated with AD and temporal lobe epilepsy [[17](#_ENREF_17),[18](#_ENREF_18)].

The interleukin 6 receptor (IL6R) is a protein encoded by the *IL6R* gene (1q21). Interleukin 6 is a potent pleiotropic pro-inflammatory cytokine that regulates cell growth and differentiation and plays an important role in the immune response. The IL6 receptor is a protein complex consisting of this protein and interleukin 6 signal transducer (IL6ST/GP130/IL6-beta), and a receptor subunit also shared by many other cytokines. IL6R may also play a role in hippocampal neurogenesis [[19](#_ENREF_19)].

1. Dorr S, Lechtenbohmer N, Rau R, Herborn G, Wagner U, et al. (2004) Association of a specific haplotype across the genes MMP1 and MMP3 with radiographic joint destruction in rheumatoid arthritis. Arthritis research & therapy 6: R199-207.

2. Deryugina EI, Quigley JP (2006) Matrix metalloproteinases and tumor metastasis. Cancer metastasis reviews 25: 9-34.

3. Szyllo K, Smolarz B, Romanowicz-Makowska H, Niewiadomski M, Kozlowska E, et al. (2002) The promoter polymorphism of the matrix metalloproteinase 3 (MMP-3) gene in women with ovarian cancer. Journal of experimental & clinical cancer research : CR 21: 357-361.

4. Beyzade S, Zhang S, Wong YK, Day IN, Eriksson P, et al. (2003) Influences of matrix metalloproteinase-3 gene variation on extent of coronary atherosclerosis and risk of myocardial infarction. Journal of the American College of Cardiology 41: 2130-2137.

5. Ghilardi G, Biondi ML, DeMonti M, Turri O, Guagnellini E, et al. (2002) Matrix metalloproteinase-1 and matrix metalloproteinase-3 gene promoter polymorphisms are associated with carotid artery stenosis. Stroke; a journal of cerebral circulation 33: 2408-2412.

6. Hinoda Y, Okayama N, Takano N, Fujimura K, Suehiro Y, et al. (2002) Association of functional polymorphisms of matrix metalloproteinase (MMP)-1 and MMP-3 genes with colorectal cancer. International journal of cancer Journal international du cancer 102: 526-529.

7. Kara I, Ozkok E, Aydin M, Orhan N, Cetinkaya Y, et al. (2007) Combined effects of ACE and MMP-3 polymorphisms on migraine development. Cephalalgia : an international journal of headache 27: 235-243.

8. Hinojosa AE, Garcia-Bueno B, Leza JC, Madrigal JL (2011) CCL2/MCP-1 modulation of microglial activation and proliferation. J Neuroinflammation 8: 77.

9. Banisadr G, Gosselin RD, Mechighel P, Kitabgi P, Rostene W, et al. (2005) Highly regionalized neuronal expression of monocyte chemoattractant protein-1 (MCP-1/CCL2) in rat brain: evidence for its colocalization with neurotransmitters and neuropeptides. J Comp Neurol 489: 275-292.

10. Semple BD, Kossmann T, Morganti-Kossmann MC (2010) Role of chemokines in CNS health and pathology: a focus on the CCL2/CCR2 and CXCL8/CXCR2 networks. J Cereb Blood Flow Metab 30: 459-473.

11. McManus C, Berman JW, Brett FM, Staunton H, Farrell M, et al. (1998) MCP-1, MCP-2 and MCP-3 expression in multiple sclerosis lesions: an immunohistochemical and in situ hybridization study. J Neuroimmunol 86: 20-29.

12. Kim JS, Gautam SC, Chopp M, Zaloga C, Jones ML, et al. (1995) Expression of monocyte chemoattractant protein-1 and macrophage inflammatory protein-1 after focal cerebral ischemia in the rat. J Neuroimmunol 56: 127-134.

13. Wang X, Feuerstein GZ (1995) Induced expression of adhesion molecules following focal brain ischemia. J Neurotrauma 12: 825-832.

14. Glabinski AR, Balasingam V, Tani M, Kunkel SL, Strieter RM, et al. (1996) Chemokine monocyte chemoattractant protein-1 is expressed by astrocytes after mechanical injury to the brain. J Immunol 156: 4363-4368.

15. Ishizuka K, Kimura T, Igata-yi R, Katsuragi S, Takamatsu J, et al. (1997) Identification of monocyte chemoattractant protein-1 in senile plaques and reactive microglia of Alzheimer's disease. Psychiatry Clin Neurosci 51: 135-138.

16. Simpson JE, Newcombe J, Cuzner ML, Woodroofe MN (1998) Expression of monocyte chemoattractant protein-1 and other beta-chemokines by resident glia and inflammatory cells in multiple sclerosis lesions. J Neuroimmunol 84: 238-249.

17. El Khoury J, Luster AD (2008) Mechanisms of microglia accumulation in Alzheimer's disease: therapeutic implications. Trends in pharmacological sciences 29: 626-632.

18. van Gassen KL, de Wit M, Koerkamp MJ, Rensen MG, van Rijen PC, et al. (2008) Possible role of the innate immunity in temporal lobe epilepsy. Epilepsia 49: 1055-1065.

19. Campbell IL, Erta M, Lim SL, Frausto R, May U, et al. (2014) Trans-signaling is a dominant mechanism for the pathogenic actions of interleukin-6 in the brain. J Neurosci 34: 2503-2513.
